# Resolving Clinically Indeterminate Findings During Anal Cancer Surveillance with TTMV-HPV DNA

**DOI:** 10.3390/cancers18010035

**Published:** 2025-12-22

**Authors:** Rafi Kabarriti, Shane Lloyd, James Jabalee, Laurie M. Gay, Tyler Slater, Kayleen Guzman, Corbin Jacobs, Sean Inocencio, Ray Lin, Cammie Nguyen, Iain MacEwan, Alexandra H. Crawford, Michael Rutenberg, Jaswinder Singh, Jennifer Ross, Sophia Kim-Wang, Chance Matthiesen, Kasha Neff, Gene-Fu Liu, Tiffany M. Juarez, Stanley L. Liauw

**Affiliations:** 1 Montefiore Medical Center and Albert Einstein College of Medicine, Bronx, NY 10467, USA; 2 Huntsman Cancer Institute, University of Utah Health, Salt Lake City, UT 84113, USA; 3 Naveris, Inc., Waltham, MA 02451, USA; 4 Cancer Care Northwest, Spokane Valley, WA 99216, USA; 5 Scripps Health, San Diego, CA 92121, USA; 6 California Protons Cancer Therapy Center, San Diego, CA 92121, USA; 7 Mayo Clinic, Jacksonville, FL 32224, USA; 8 MidAmerica Cancer Care, LLC, Kansas City, MO 64132, USA; 9 Department of Radiation and Cellular Oncology, University of Chicago, Chicago, IL 60637, USA; 10 Freeman Radiation Oncology, Joplin, MO 64804, USA; 11 Providence Mission Hospital, Mission Viejo, CA 92691, USA

**Keywords:** TTMV-HPV DNA, HPV, ASCC, post-treatment, surveillance, anal cancer, ctDNA, circulating tumor DNA

## Abstract

Background/Objectives: Surveillance for anal squamous cell carcinoma (ASCC) recurrence relies on clinical examination and imaging. Post-treatment edema, fibrosis, and inflammation can result in clinically indeterminate findings (CIFs) that delay diagnosis and increase patient and system burden. Circulating tumor tissue-modified viral (TTMV)-HPV DNA offers a biologically specific, noninvasive biomarker that may clarify equivocal assessments. Methods: In this multi-center retrospective study, 233 patients with HPV-associated ASCC were evaluated, including 185 with ≥1 post-treatment TTMV-HPV DNA test. CIFs were defined as exams or imaging results not definitively positive or negative for disease, and were paired with subsequent TTMV-HPV DNA tests. Concordance was defined by prespecified follow-up windows comparing TTMV-HPV DNA results with subsequent clinical outcomes. Results: Ninety patients (39%) experienced 214 CIFs, arising from exams (46%, 98) or imaging (54%, 116). Indeterminate rates by assessment were 7% for exams, 17% for imaging, and 1.3% for TTMV-HPV DNA testing. Overall, 52 CIF/TTMV-HPV DNA pairs were eligible for analysis, and TTMV-HPV DNA resolved disease status accurately for 48/52 (92%, 95% CI: 81.5–97.9). Negative tests predicted cancer-free status for 37/41 CIFs (90%), while 100% of positive tests (11/11) were concordant with clinically confirmed recurrence. In 73% of positive cases (8/11), TTMV-HPV DNA was the first indication of recurrence (median lead-time 29 days; IQR 25–147). Conclusions: TTMV-HPV DNA testing reliably clarifies clinically indeterminate findings during ASCC surveillance, demonstrating high accuracy (92%) and earlier detection of recurrence. These data support integration into post-treatment management to reduce diagnostic uncertainty and guide timely care.

## Simple Summary

During post-treatment surveillance for anal squamous cell carcinoma (ASCC), clinical examination, anoscopy, and imaging frequently yield inconclusive findings that cannot be confidently classified as the presence or absence of disease. These clinically indeterminate findings (CIFs) delay care decisions and lead to additional procedures that carry financial, physical, and psychological burden. Circulating tumor tissue-modified viral (TTMV)-HPV DNA measured from plasma is accessible, repeatable, and independent of local post-treatment anatomic changes. In this multi-center retrospective cohort, TTMV-HPV DNA testing accurately resolved 92% of CIFs and positive tests often served as the earliest indicator of recurrence. These findings support the clinical utility of TTMV-HPV DNA to clarify indeterminate assessments post-treatment and help guide next steps in care.

## 1. Introduction

Human papillomavirus (HPV) underlies the vast majority of the estimated 11,000 annual new cases of anal squamous cell carcinoma (ASCC) in the United States [1,2,3]. Despite curative-intent chemoradiotherapy (CRT), up to 30% of patients experience persistent disease after treatment or recurrence [4,5,6]. Patterns of regression after CRT are highly variable, and HPV-positive tumors may continue to involute slowly for months in a phenomenon that has been well-described [7,8].

Standard surveillance begins 8–12 weeks after primary treatment, and includes visual exam, digital anorectal examination, and inguinal lymph node evaluation, with ongoing exams every 3–6 months for 5 years, plus biannual anoscopy and annual CT and MRI for 3 years [9]. For patients without complete clinical response, closer follow-up over the ensuing 6 months is recommended to distinguish regression from residual malignant disease.

In practice, however, the accuracy of surveillance examinations is constrained by many factors. Post-treatment edema, inflammation, and pain can obscure early recurrence and yield clinically indeterminate findings (CIFs). Limited fields of view, slow tumor regression and delayed healing may also present challenges. For these reasons, initial post-treatment imaging is often delayed up to 26 weeks [9,10,11]. The diagnostic accuracy of anoscopy also varies by operator experience and is not broadly available [12]. Finally, post-treatment fibrosis and hemorrhoids can present similarly to cancer recurrence, and vice versa. These limitations create significant clinical uncertainty, often leading to repeated evaluations, additional procedures, and patient anxiety.

Given these challenges, there remains a critical need for an accessible, reliable test independent of local anatomic changes to help differentiate residual or recurrent disease from benign post-treatment effects. In this work, we investigated the role of circulating tumor tissue modified viral (TTMV)-HPV DNA as a candidate biomarker to offer a tumor-specific and anatomically unrestrained surveillance tool. In prior work, Kabarriti et al. demonstrated high specificity (98.4%) and strong positive (96.0%) and negative (92.5%) predictive values for recurrence detection using TTMV-HPV DNA, which accurately resolved 94.3% of CIFs [13]. The present multi-center retrospective study expands on earlier work to further examine the utility of TTMV-HPV DNA testing for resolving CIFs encountered during routine ASCC surveillance. We examined the frequency and timing of CIFs, compared indeterminate rates across surveillance modalities, and assessed the accuracy of the TTMV-HPV DNA in predicting near-term disease status when standard assessments were inconclusive.

## 2. Materials and Methods

### 2.1. Study Design and Oversight

This multi-center retrospective clinical case series was conducted across ten participating U.S. centers with data-sharing and Institutional Review Board (IRB) approvals. A central independent IRB (WCG IRB, Cary, NC, USA) granted a waiver of written informed consent. The study adhered to site policies and recognized ethical standards.

### 2.2. Participants and Setting

The retrospective cohort of patients with biopsy-confirmed HPV-associated ASCC were eligible if they underwent at least one TTMV-HPV DNA test during routine clinical care between January 2020 and February 2025. Tumor HPV status was determined using p16 immunohistochemistry (IHC) and/or molecular assays, including TTMV-HPV DNA (blood or tissue), HPV polymerase chain reaction (PCR), or HPV RNA in situ hybridization (ISH). A pretreatment TTMV-HPV DNA test was not required.

### 2.3. Data Sources and Variables

Trained research personnel at each site abstracted a limited, de-identified dataset into a centralized electronic database. Variables included demographics (sex as a biological variable, race/ethnicity, smoking history, HIV status, transplant status, and cancer diagnosis date), tumor characteristics (primary site, stage, HPV testing method and result, and HPV genotype, if available), treatments (modality and start/end dates), and clinical assessments (date, modality, and outcome for exams, imaging, and biopsies). Clinical outcomes were coded as “active disease”, “indeterminate or suspicious”, or “no evidence of disease” (NED) based on clinical documentation. Vital status (alive or deceased, and date of death or last follow-up) was also collected.

### 2.4. Endpoints

The primary endpoint was concordance of TTMV-HPV DNA with near-term clinical outcome for CIF-associated tests, expressed as the proportion of CIF/TTMV-HPV DNA pairs correctly predicting presence or absence of disease within the prespecified windows. Secondary endpoints included the proportion of positive TTMV-HPV DNA tests that preceded clinical detection of recurrence (lead time; days), the rate of indeterminate TTMV-HPV DNA results compared with indeterminate rates for exams and imaging, and descriptive timing of CIF occurrence relative to completion of primary treatment.

### 2.5. Definition of CIFs and Pre-Specified Criteria

CIFs were defined a priori as post-treatment exam or imaging assessments performed during routine care that were classified as indeterminate because they could not be categorized as definitively positive or negative for disease. Each assessment was evaluated independently such that its classification did not depend on the results of other assessments. Because patients can undergo multiple assessments, they could experience multiple CIFs. Only assessments obtained after completion of primary treatment and before initiation of salvage therapy were included.

### 2.6. Pairing of CIFs with TTMV-HPV DNA Tests 

To evaluate concordance of TTMV-HPV DNA with near-term clinical outcomes, each CIF was paired with the nearest subsequent TTMV-HPV DNA test obtained within 3 months. This interval reflects typical NCCN-concordant surveillance in the first 2 years after treatment and the follow-up timing for equivocal findings (Figure S1A). Each TTMV-HPV DNA test was uniquely paired to a single CIF.

### 2.7. Outcome Determination and Follow-Up Windows

Prespecified follow-up windows were used to adjudicate concordance between TTMV-HPV DNA results and clinical outcomes. Recurrence was defined as any exam, imaging study, or biopsy demonstrating active disease. Negative TTMV-HPV DNA results were considered concordant if no recurrence occurred before or within 3 months after the test, and discordant if recurrence occurred within that window. Positive TTMV-HPV DNA results were considered concordant if clinical recurrence was clinically documented at any time before or after the test. Positive tests without recurrence were only considered discordant if ≥6 months of follow-up was available. Tests lacking sufficient follow-up (≥3 months for concordant negatives and ≥6 months for discordant positives) were censored. One test was censored a priori because a biopsy performed 41 days before blood collection may have removed the lesion and may have altered TTMV-HPV DNA detectability. Indeterminate TTMV-HPV DNA tests were excluded from CIF/TTMV-HPV DNA pairing. The complete pairing, censoring, and outcome rubric is depicted in Figure S1B,C.

### 2.8. TTMV-HPV DNA Testing

Peripheral blood samples collected in DNA stabilizing tubes (Streck Inc., La Vista, NE, USA) per manufacturer guidelines were sent to the Naveris clinical laboratory for testing with the commercially available NavDx^®^ assay (Naveris, Inc., Waltham, MA, USA), as previously described [13,14]. NavDx employs droplet digital PCR with algorithmic analysis of fragmentation patterns to detect tumor-derived HPV DNA from five high-risk subtypes (HPV-16, -18, -31, -33, and -35). Results are reported as negative, indeterminate, or positive, with quantitative TTMV-HPV DNA scores provided. The range for indeterminate results differed by genotype and date (HPV16: 5–12 before 13 June 2022, and 2–3 after that date; other HPV genotypes: 5–12 for all), and are pre-defined analytical thresholds established by Naveris, Inc. (Waltham, MA, USA). For positive results, detected HPV genotype(s) are reported.

### 2.9. Statistical Analysis

Continuous variables were summarized using the mean and standard deviation or median and interquartile range. Categorical variables were summarized as counts and percentages. Exact binomial 95% confidence intervals were calculated for concordance and modality-specific indeterminate rates. Two-sided *p*-values < 0.05 were considered statistically significant. Analyses were conducted using Python (version 3.12).

## 3. Results

### 3.1. Study Population

A total of 233 patients with HPV-associated ASCC were included across 10 U.S. centers, including longitudinal updates for 117 patients (50%) analyzed previously [13]. At least one post-treatment exam, imaging study, or biopsy was performed for 215 patients, of which 90 patients (42%) had ≥1 CIF.

Patient demographics and tumor characteristics are outlined in Table 1. Most patients were female (74%) and White (75%), with a mean age of 64 years (range: 30–91). HPV status was confirmed by p16 IHC alone for 193 patients (83%), whereas 15 (7%) had PCR or ISH, with or without other methods. The remaining patients had their HPV status confirmed by TTMV-HPV DNA (tissue or blood, *N* = 24, 10%) or by an unrecorded method (*N* = 1, 0.4%). Most tumors were staged T2-T3 (*N* = 154, 66%), N0-N1 (*N* = 220, 99%), and M0 (*N* = 217, 93%). Concurrent CRT was the predominant treatment approach (*N* = 200, 86%).

While not required for study inclusion, pretreatment TTMV-HPV DNA testing was available for 123 patients (53%). In this subcohort, HPV status was established by p16 IHC in 91/123 (74%), by PCR and/or ISH with or without other methods in 9/123 (7%) and exclusively by TTMV-HPV DNA (blood or tissue) in 23/123 (19%). Of the 123 tested patients, 106 tested positive for pretreatment TTMV-HPV DNA (pretreatment sensitivity: 86.2%, 95% CI: 78.8–91.7).

There were 603 total post-treatment TTMV-HPV DNA tests across 185 patients. Successive TTMV-HPV DNA testing occurred at a median interval of 3.7 months (IQR: 3.0–6.2), reflecting a similar cadence to NCCN-recommended surveillance. Test performance metrics are summarized in Table S1.

### 3.2. Frequency and Timing of CIFs and Indeterminate TTMV-HPV DNA Tests

**Figure 1:**
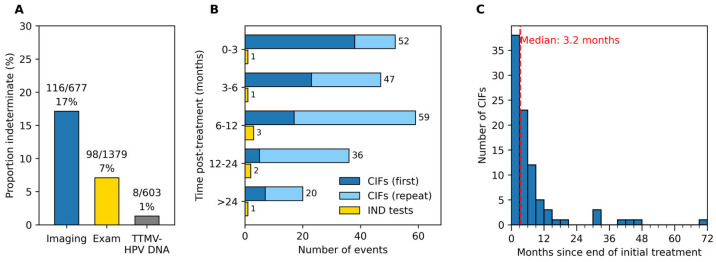
Frequency and timing of clinically indeterminate findings (CIFs) and indeterminate TTMV-HPV DNA tests after completion of initial treatment. (**A**) Proportion of post-treatment assessments that were indeterminate, stratified by modality. (**B**) Counts of CIFs and indeterminate TTMV-HPV DNA tests by surveillance stage. Surveillance stages were left-inclusive to avoid overlap at bin boundaries. Dark blue bars indicate the patient’s first CIF. Light blue bars indicate repeat CIFs or TTMV-HPV DNA tests. (**C**) Distribution of time in months from the end of initial treatment to each patient’s first CIF (3-month bins); red dashed line indicates the median.

Of 215 patients with ≥1 post-treatment exams and/or imaging studies, 90 (42%) had ≥1 post-treatment CIF(s). Among these 90 patients, 214 CIFs were observed, with 46% (98/214) arising from clinical examination and 54% (116/214) from imaging. When normalized to the total number of assessments, CIFs occurred in 17% (116/677) of imaging studies and 7% (98/1379) of exams (Figure 1A; Table 2). Patients with CIFs experienced a median of 2 (range: 1–8); 59% (53/90) had multiple CIFs, with 32 patients having sequential indeterminate assessments. Those with sequential CIFs had a median interval of 42 days between their first and last inconclusive assessment (IQR: 12–162) and 80 days (IQR: 39–148) between their first indeterminate result and a conclusive follow-up. In contrast, only 4.3% (8/185) of patients with post-treatment TTMV-HPV DNA testing had an indeterminate result, representing 1.3% (8/603) of all TTMV-HPV DNA tests (Figure 1A, Table 2). No patient had more than 1 indeterminate TTMV-HPV DNA result. 

Of those patients with ≥1 year of clinical follow-up, 53% (63/119) experienced a CIF, compared to 59% (39/66) for patients with ≥2 years of clinical follow-up. The indeterminate rate remained stable during the first year after treatment, with comparable frequencies across the 0–3, 3–6, and 6–12 month intervals, but declined beyond 12 months (Figure 1B; Table 2). CIFs occurred most often between 6 and 12 months post-treatment (59/214, 28%), followed by 0–3 months (52/214, 24%), 3–6 months (47/214, 22%), 12–24 months (36/214, 17%), and ≥24 months (20/214, 9%). The median time from completion of treatment to the first CIF was 97 days (IQR: 52–204) (Figure 1C). The odds of having a CIF within the first year post-treatment were 1.86 times higher than those in the second year (95% CI: 1.23–2.80; Fisher’s exact test, *p* = 0.0026). 

A patient’s first indeterminate TTMV-HPV DNA test occurred a median of 288 days post-treatment (IQR: 178–414). Indeterminate TTMV-HPV DNA tests occurred at a rate of 5/301 (0.017) in year 1 and 2/162 (0.012) in year 2.

### 3.3. Resolution of Indeterminate Disease Status by TTMV-HPV DNA Testing

To evaluate the performance of TTMV-HPV DNA to resolve indeterminate clinical disease status, CIFs were paired with TTMV-HPV DNA tests obtained within a 3-month window after the CIF. Each TTMV-HPV DNA result was then compared with subsequent clinical follow-up and classified as correctly or incorrectly predicting recurrence or the absence of recurrence in the 3 months following the test (Figure S1B,C).

**Figure 2:**
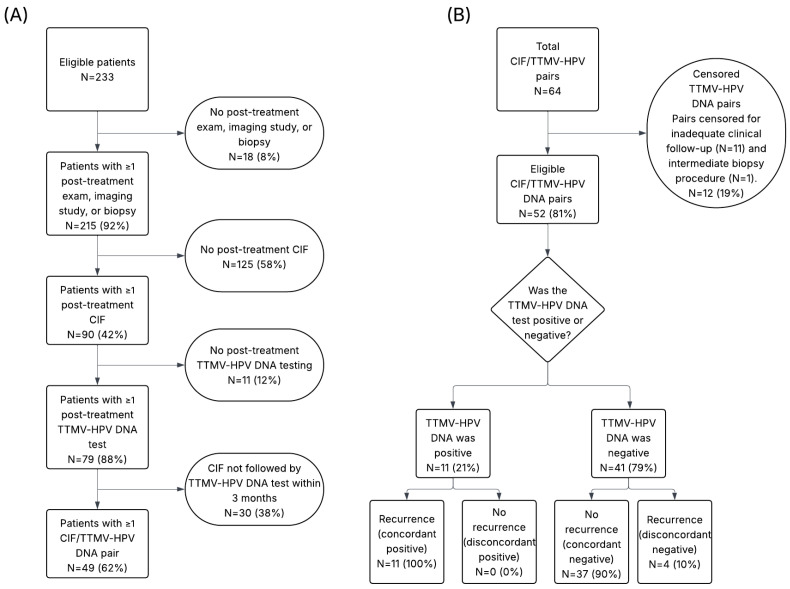
STARD chart depicting the flow of patients and CIF/TTMV-HPV DNA pairs through study inclusion and analysis. (**A**) Patient inclusion flow diagram. The total cohort included all study-eligible patients, of whom a subset underwent post-treatment TTMV-HPV DNA testing. Of these, patients with ≥1 CIF were identified, and those with a CIF occurring within 3 months before a TTMV-HPV DNA test were included as CIF/TTMV-HPV DNA pairs. (**B**) Flow of CIF/TTMV-HPV DNA pairs through censoring and classification. Pairs were excluded if follow-up was insufficient or if an intermediate biopsy interfered with TTMV-HPV DNA levels. The remaining pairs were classified as concordant or discordant based on recurrence status within the defined follow-up window. Percentages represent the proportion of patients within each step of the flow diagram relative to the group in the preceding row.

**Figure 3:**
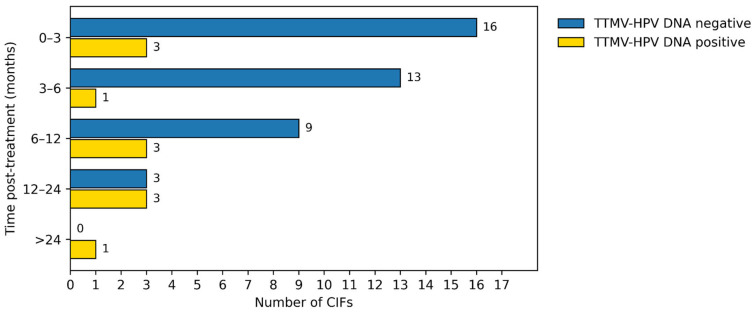
Distribution of CIF/TTMV-HPV DNA pairs by time post-treatment, stratified by TTMV-HPV DNA result. Bars represent the number of CIF/TTMV-HPV DNA pairs occurring in each surveillance interval.

**Figure 4:**
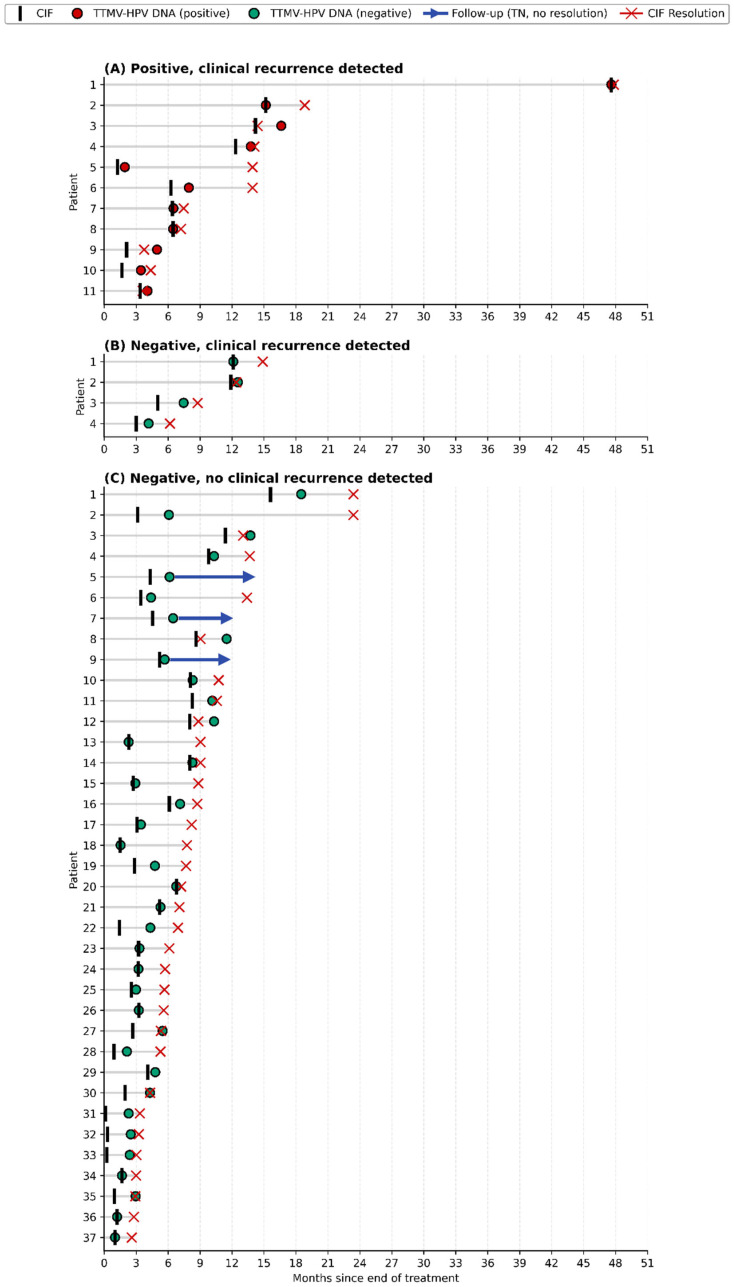
Classification of CIFs by paired TTMV-HPV DNA test result and subsequent clinical outcome. Each horizontal line represents a CIF/TTMV-HPV DNA pair, with time zero marking the end of primary treatment. A vertical black bar indicates the CIF, and each colored circle represents the paired TTMV-HPV DNA test (red = positive, green = negative). A red X indicates resolution of the CIF to a definitive clinical status. In cases where clinical resolution is not achieved, a blue arrow extends to the end of clinical follow-up. Panels show clinically relevant subsets: (**A**) Positive TTMV-HPV DNA, clinical recurrence detected (positive concordant). (**B**) Negative TTMV-HPV DNA, clinical recurrence detected (negative discordant). (**C**) Negative TTMV-HPV DNA, no clinical recurrence detected (negative concordant).

Of 214 CIFs, 64 (30%) were paired with a TTMV-HPV DNA test obtained within 3 months. After excluding one pair due to a potentially lesion-altering biopsy and 11 pairs with insufficient follow-up, 52 CIF/TTMV-HPV DNA pairs were evaluable (Figure 2). Most pairs occurred within the first 6 months post-treatment (33/52, 63%; Figure 3). Longitudinal follow-up for each evaluable CIF/TTMV-HPV DNA pair is shown in Figure 4.

Positive TTMV-HPV DNA tests occurred in 21% (11/52) of evaluated pairs. All positive tests were concordant with clinically confirmed recurrence (100% concordance). TTMV-HPV DNA testing was the first indication of recurrence in 73% (8/11) of these cases, with a median lead-time of 29 days (IQR: 19–129). In 75% (6/8) of instances in which TTMV-HPV DNA was the first indication of recurrence, the recurrence was detected within 6 months after testing. In 3 cases, the CIF-associated TTMV-HPV DNA test confirmed clinical recurrence detection.

Negative TTMV-HPV DNA results occurred in 79% (41/52) of evaluable pairs. Of these, 90% (37/41) were concordant with the absence of recurrence within the prespecified follow-up window. In 4 instances (4/41, 10%) negative tests were discordant and were associated with clinically confirmed locoregional recurrence (Figure 2B). Of those patients, 2 had low pretreatment TTMV-HPV DNA test scores (23 and 86; HPV16), and both had biopsy-proven, p16-positive recurrences. Biopsies were performed 61 and 84 days after negative testing, respectively. The other 2 patients were not tested for pretreatment TTMV-HPV DNA. The first patient had biopsy-proven, p16-positive recurrence. The second patient’s recurrence was diagnosed via sigmoidoscopy performed 40 days after the negative TTMV-HPV DNA test and was not biopsy-confirmed.

Overall, TTMV-HPV DNA testing correctly predicted the disease status for 92% of CIF-associated tests (48/52, 95% CI: 82–98; Figure 2B). TTMV-HPV DNA correctly predicted disease status for 96% (26/27) of CIFs performed within 4 months after treatment, and 88% (22/25) of CIFs performed after 4 months post-treatment.

### 3.4. Recurrence Detection Following Positive TTMV-HPV DNA Testing

Among patients undergoing post-treatment surveillance, discordance occasionally occurred when a positive TTMV-HPV DNA test was obtained before any clinical evidence of disease. Across the cohort, 30 positive tests were recorded in 11% of patients (21/185). In every case, the positive test served as the first indication of recurrence. At the time of each patient’s initial positive test, clinical status was recorded as “NED” in 57% (12/21), “indeterminate” in 24% (5/21), and “unknown” in 19% (4/21).

**Figure 5:**
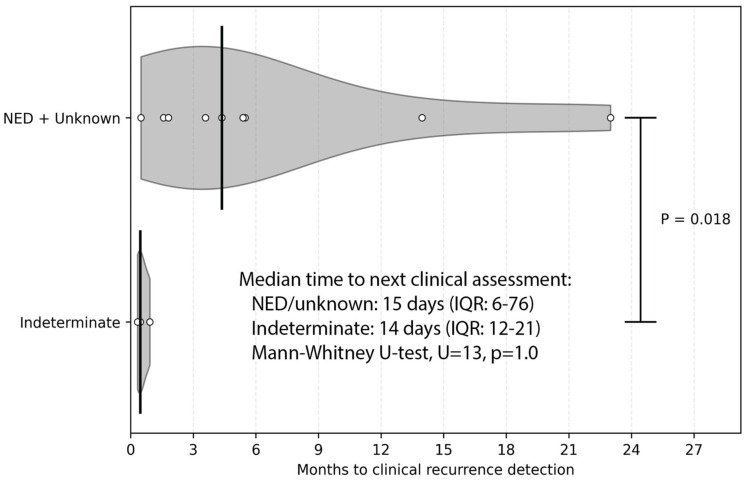
Time to clinical recurrence detection following a positive TTMV-HPV DNA test by clinical status at the time of testing. Violin plots show the distribution of time to clinical recurrence detection in months for patients with indeterminate clinical status vs. no evidence of disease (NED) or unknown status at the time of testing. Individual observations are overlaid (white circles) and vertical lines indicate group medians. Patients with NED or unknown clinical status took longer to have their clinical recurrence detected than those with indeterminate status (Mann–Whitney U-test, U = 1, *p* = 0.018).

Eight patients lacked sufficient follow-up (≥6 months) to determine clinical correlation. Among the remaining 13 patients, recurrence was eventually confirmed in all cases (100%). Recurrence was identified more quickly when the paired clinical assessment was indeterminate compared with NED or unknown status (median 14 days vs. 133 days, Mann–Whitney U-test, U = 1, *p* = 0.018; Figure 5). To confirm that this difference was not driven by unequal follow-up cadence, we also evaluated the time from the positive TTMV-HPV DNA test to the next clinical assessment. The median time to the next assessment did not differ by disease status (14 days vs. 15 days), but the range was larger for patients with “unknown” or “NED” status (4–120 days) compared to those with “indeterminate” status (10–28 days). Overall, only 8 of 185 patients (4.3%) had unresolved positive tests due to limited follow-up, reflecting strong concordance between positive TTMV-HPV DNA results and clinical recurrence.

## 4. Discussion

Surveillance for ASCC is complicated by post-treatment anatomic and inflammatory changes, which frequently produce CIFs and delay diagnostic clarity. In this multi-center cohort, TTMV-HPV DNA testing resolved the vast majority of CIFs and often anticipated clinical confirmation of recurrence by several weeks. Importantly, the test demonstrated a very low indeterminate rate (1.3%) compared with clinical examination and imaging, reinforcing its value in situations where traditional methods are limited.

Clinical guidelines call for intensive anal cancer post-treatment assessment and surveillance, which include physical examination, CT, MRI, and/or PET/CT [9,16,17,18]. Each of these modalities has limitations in identifying recurrent or residual disease, with positive predictive values (PPV) for MRI, anoscopy, and digital anorectal exam ranging from 30% to 60% [19,20,21,22].

The limitations inherent in these surveillance modalities lead to clinical uncertainty. Many patients continue to show tumor regression and eventual resolution up to 6 months post treatment, with significant ongoing changes at the primary tumor site complicating visualization [11]. In addition, beginning post-treatment surveillance with imaging or physical examination can be limited by CRT-induced physical changes at the site of the primary tumor [23,24,25], including pain, fibrosis, and inflammation. It is unsurprising that ambiguous clinical examinations and diagnostic testing can place a significant psychological toll on patients, leading to increased anxiety [26,27,28,29,30,31,32].

A multi-modal approach to surveillance reduces uncertainty. Several studies illustrate the complementary nature of physical examination and imaging. Houard et al. reported that up to 10% of patients in one study had locoregional recurrence detected by PET/CT that was not observed with clinical examination [33]. Cutting through the uncertainty in CIFs in a timely fashion allows detection when the extent of disease is still limited, leading to the best outcomes from salvage surgical treatment [33,34,35,36].

The use of liquid biopsy assays can complement existing tools and overcome limitations of imaging and physical examination to detect cancer outside the field of view, lesions obscured by fibrosis or inflammation, or tumors smaller than the detection limits [13,37,38,39]. Within this cohort of patients with anal cancer, more than 40% experienced ≥1 CIF post-treatment, with 46% of CIFs occurring within the first 6 months post-treatment. In addition, nearly 25% of all patients experienced multiple post-treatment CIFs, with 14% of all patients having sequential indeterminate assessments, with a median of 80 days between the first inconclusive result and a conclusive confirmation of disease status. By comparison, TTMV-HPV DNA testing had an overall indeterminate rate of 1.3%, compared to 17% from imaging and 7% from clinical examination. The clinical validity of circulating tumor DNA in the post-treatment setting for ASCC has been shown repeatedly, with sensitivities greater than 85% [5,40,41,42,43,44]. TTMV-HPV DNA has demonstrated a per-patient PPV for recurrent disease of 96.0% and a point-in-time NPV of 92.5% [13]. Consistent with previous studies [13,39], TTMV-HPV DNA detection via liquid biopsy provides an accurate method for resolving post-treatment CIFs in patients with anal cancer. In this follow-on study to Kabarriti et al., more than 90% of TTMV-HPV DNA tests were concordant with subsequent exams or scans. These metrics suggest that patients with a CIF and negative TTMV-HPV DNA results, 90% (37/41) in this cohort, can confidently be considered recurrence-free. This high rate of accuracy can reassure patients of the response, as well as minimize unnecessary interventions and the accompanying risk for adverse events. Although less frequent, recurrent disease at the time of a CIF was confirmed in 100% of patients who had a paired-positive TTMV-HPV DNA test.

Providing additional information with a liquid biopsy assay to resolve clinical ambiguity has the potential to save both time and money compared to repeat procedures or imaging. With sample acquisition possible by clinic-based or mobile phlebotomy and a turn-around time of 1 week, testing for plasma TTMV-HPV DNA offers a way to substantially shorten the period of ambiguity and quickly define next steps. Access to mobile phlebotomy or local clinics can reduce the burden of both time and cost for patients, with improved equity in care. This study has limitations inherent to its retrospective design. Anal cancer is a rare disease, and the current cohort represents approximately 2% of the annual incidence. Patients were unselected and represented a broad population treated at ten geographically dispersed sites. Surveillance intervals and testing timing were not standardized, and clinicians may have been more likely to order TTMV-HPV DNA testing for patients with concerning findings, potentially enriching for patients with CIFs and potentially increasing the apparent clinical utility in this population. The small number of patients who did not receive TTMV-HPV DNA testing in surveillance precludes a robust comparison of CIFs between groups. Further, although positive test results are likely to prompt follow-up, this behavior could not be systematically captured. Finally, most tumors (83%) were assessed by p16 IHC, which may misclassify patients as HPV-positive 79% to 95% of the time [45,46,47], as p16 positivity does not always indicate transcriptionally active HPV, potentially affecting the accuracy of the TTMV-HPV DNA test interpretations.

Despite these limitations, this study represents one of the largest multi-center evaluations of CIFs in ASCC and provides robust evidence supporting the clinical utility of TTMV-HPV DNA testing in resolving indeterminate surveillance findings.

## 5. Conclusions

TTMV-HPV DNA assessment by liquid biopsy is a valuable adjunct to standard ASCC surveillance, offering high accuracy, low indeterminate rates, and early detection of recurrence. By reliably resolving clinically indeterminate findings, TTMV-HPV DNA provides actionable information that can accelerate appropriate diagnostic workup or reassure patients when no recurrence is present. These findings support its integration into routine post-treatment follow-up, particularly in the early months after CRT when CIFs are most prevalent and traditional surveillance modalities are most limited. This approach may reduce diagnostic delay, decrease unnecessary procedures, and lessen patient anxiety associated with prolonged uncertainty.

## Figures and Tables

**Table 1:** Patient and tumor characteristics.

Characteristic	All Patients *N* = 233 (%)	Patients with CIF(s) *N* = 90 (%)	Patients with Post-Treatment TTMV-HPV DNA *N* = 185 (%)
Mean ± SD age (range), years	64 ± 11 (30–91)	63 ± 12.2 (36–88)	64 ± 11 (30–88)
Biological sex			
Female	173 (74.2)	60 (66.7)	142 (76.8)
Male	59 (25.3)	29 (32.2)	42 (22.7)
Intersex	1 (0.4)	1 (1.1)	1 (0.5)
Race and ethnicity			
White	174 (74.7)	64 (71.1)	135 (73.0)
Black	18 (7.7)	11 (12.2)	14 (7.6)
American Indian or Alaska Native White	2 (0.9)	1 (1.1)	2 (1.1)
Native Hawaiian or Other Pacific Islander	2 (0.9)	0 (0)	2 (1.1)
Asian	1 (0.4)	0 (0)	0 (0)
Other	22 (9.4)	7 (7.8)	19 (10.3)
Unknown	14 (6.0)	7 (7.8)	13 (7.0)
Smoking status			
Never	122 (52.4)	47 (52.2)	96 (51.9)
Former	74 (31.8)	29 (32.2)	59 (31.9)
Current	30 (12.9)	13 (14.4)	24 (13.0)
Unknown	7 (3.0)	1 (1.1)	6 (3.2)
HPV reference testing method			
p16 IHC	193 (82.8)	77 (85.6)	157 (84.9)
TTMV-HPV DNA (blood)	21 (9.0)	4 (4.4)	11 (5.9)
p16 IHC and HPV PCR or ISH	9 (3.9)	4 (4.4)	8 (4.3)
HPV PCR or ISH	6 (2.6)	4 (4.4)	5 (2.7)
TTMV-HPV DNA (tissue)	3 (1.3)	1 (1.1)	3 (1.6)
Method not recorded	1 (0.4)	0 (0)	1 (0.5)
HPV subtype ^A^			
16	118 (91.5)	40 (90.9)	93 (94.9)
18	3 (2.3)	1 (2.3)	1 (1.0)
31	4 (3.1)	2 (4.5)	2 (2.0)
33	3 (2.3)	1 (2.3)	1 (1.0)
35	1 (0.8)	0 (0)	1 (1.0)
Initial primary tumor staging ^B,C^			
T0	1 (0.4)	1 (1.1)	1 (0.5)
T1	40 (17.2)	13 (14.4)	33 (17.8)
T2	91 (39.1)	35 (38.9)	78 (42.2)
T3	63 (27.0)	26 (28.9)	50 (27.0)
T4	31 (13.3)	14 (15.6)	19 (10.3)
Tx	7 (3.0)	1 (1.1)	4 (2.2)
Nodal status at baseline ^B,C^			
N0	108 (46.4)	31 (34.4)	87 (47.0)
N1	112 (48.1)	53 (58.9)	91 (49.2)
N2	6 (2.6)	4 (4.4)	4 (2.2)
N3	1 (0.4)	1 (1.1)	0 (0)
Nx	6 (2.6)	1 (1.1)	3 (1.6)
Distant metastasis at baseline ^B^			
M0	217 (93.1)	89 (98.9)	181 (97.8)
M1	12 (5.2)	1 (1.1)	2 (1.1)
Mx	4 (1.7)	0 (0)	2 (1.1)
Initial treatment modality			
Concurrent CRT	200 (85.8)	76 (84.4)	164 (88.6)
Radiotherapy alone	10 (4.3)	6 (6.7)	9 (4.9)
Chemotherapy alone	7 (3.0)	2 (2.2)	4 (2.2)
Concurrent CRT + other systemic therapy	6 (2.6)	5 (5.6)	5 (2.7)
Surgery	4 (1.7)	0 (0)	1 (0.5)
Surgery + Adjuvant RT or CRT	2 (0.9)	1 (1.1)	2 (1.1)
Chemoimmunotherapy	1 (0.4)	0 (0)	0 (0)
No treatment	3 (1.2)	0 (0)	0 (0)

^A^ HPV subtype was only available for patients with genotyping from PCR, ISH, or a positive TTMV-HPV DNA test performed at any time (*N* = 129 patients, including 44 with a CIF and 98 with post-treatment TTMV-HPV DNA testing). ^B^ T, N, and M stages were derived using pathologic staging when available, and clinical staging otherwise. Staged according to the American Joint Committee on Cancer in effect at the time of diagnosis (7th, 8th, or 9th Edition, as applicable) [15]. ^C^ Sub-stages (a, b, c) are collapsed into their parent stage. Abbreviations: CIF, Clinically indeterminate finding; CRT, chemoradiation therapy; IHC, immunohistochemistry; ISH, in situ hybridization; RT, radiotherapy.

**Table 2:** Indeterminate rate by surveillance stage and assessment modality.

	Imaging			Exam			TTMV-HPV DNA		
Surveillance stage *	Ind	Total	%	Ind	Total	%	Ind	Total	%
0–3 months	22	126	17.5	30	360	8.3	1	80	1.3
3–6 months	31	132	23.5	16	229	7.0	1	88	1.1
6–12 months	34	149	22.8	25	286	8.7	3	135	2.2
12–24 months	17	159	10.7	19	288	6.6	2	167	1.2
>24 months	12	111	10.8	8	216	3.7	1	133	0.8
All stages	116	677	17.3	98	1379	7.1	8	603	1.3

* Surveillance stages were left-inclusive to avoid overlap at bin boundaries. Abbreviations: Ind, Indeterminate.

## Data Availability

The data supporting this study’s findings are available from the corresponding author upon reasonable request and subject to institutional review and data-sharing agreements.

## Supplementary Materials

The following supporting information can be downloaded at: https://www.mdpi.com/article/10.3390/cancers18010035/s1, Figure S1: Rule set for CIF/TTMV-HPV DNA pairing and outcome classification with NCCN-concordant surveillance cadence. Table S1: Post-treatment TTMV-HPV DNA metrics.

## Abbreviations

The following abbreviations are used in this manuscript:

ASCC	Anal squamous cell carcinoma
CIF	Clinically indeterminate finding
CI	Confidence interval
CRT	Chemoradiotherapy
HPV	Human papillomavirus
IHC	Immunohistochemistry
IND	Indeterminate
IQR	Interquartile range
IRB	Institutional review board
ISH	In situ hybridization
NED	No evidence of disease
NPV	Negative predictive value
PCR	Polymerase chain reaction
PPV	Positive predictive value
RT	Radiotherapy
TTMV	Tumor tissue modified viral
