# Supplementary material for: Resolving Clinically Indeterminate Findings During Anal Cancer Surveillance with TTMV-HPV DNA
Source: Cancers (Basel). 2025 Dec 22;18(1):35. doi: 10.3390/cancers18010035 (PMC12784822; doi:10.3390/cancers18010035)
Supplement: Supplementary file 1 [file cancers-18-00035-s001.zip › Supplementary Figure S1.pdf]

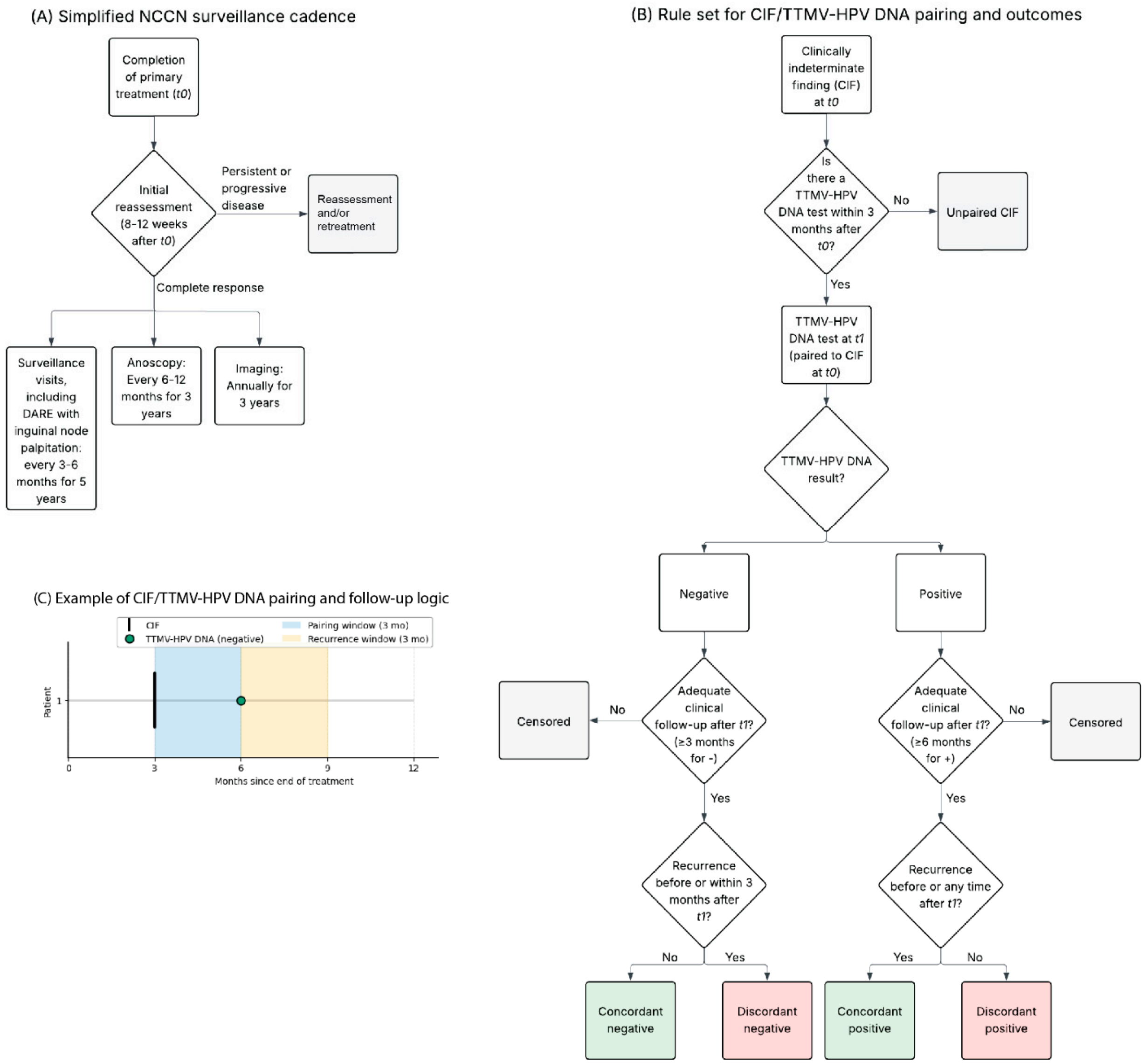

Figure S1. Rule set for CIF/TTMV-HPV DNA pairing and outcome classification with NCCN-concordant surveillance cadence. (A) Simplified NCCN-concordant surveillance after curative treatment. According to the NCCN guidelines, patients are reassessed at 8-12 weeks post-treatment, receive DARE with inguinal node exam every 3-6 months for 5 years, anoscopy every 6-12 months for 3 years, and annual imaging for 3 years. (B) Because this study was retrospective, surveillance assessments and TTMV-HPV DNA testing were performed according to routine clinical practice rather than a fixed schedule. Therefore, a standardized rule set was applied to retrospectively pair CIFs and TTMV-HPV DNA tests and to define outcomes. **Pairing:** CIFs were paired only if a TTMV-HPV DNA test occurred within 3 months after the CIF; unpaired CIFs were excluded. **Censoring:** After pairing, tests in patients without recurrence that lack sufficient follow-up were censored (<3 months for negatives, and <6 months for positives without documented recurrence). **Outcomes:** Negative tests were considered concordant if no recurrence occurred before or within 3 months post-test and discordant if recurrence occurred within that window. Positive tests were considered concordant if recurrence was documented at any time (before or after the test) and discordant if no recurrence was documented with ≥6 months of follow-up. (C) An example of pairing and follow-up logic for a negative TTMV-HPV DNA test after a CIF is depicted. A CIF (black vertical bar) is paired with a TTMV-HPV DNA test (green dot) if the test occurs within 3 months after the CIF (transparent blue box, the “pairing window”). For negative tests, an

additional 3-month "recurrence window" (gold box) is applied to determine concordance. For positive tests (not shown), the recurrence window extends through the remainder of follow-up, and the result is concordant if recurrence is documented at any time.

Abbreviations: CIF, clinically indeterminate finding; DARE: Digital anorectal exam; NCCN, National Comprehensive Cancer Network; TTMV-HPV DNA, tumor tissue-modified viral HPV DNA.
