# Supplementary material for: Resolving Clinically Indeterminate Findings During Anal Cancer Surveillance with TTMV-HPV DNA
Source: Cancers (Basel). 2025 Dec 22;18(1):35. doi: 10.3390/cancers18010035 (PMC12784822; doi:10.3390/cancers18010035)
Supplement: Supplementary file 1 [file cancers-18-00035-s001.zip › Supplementary Table S1.pdf]

Table S1. Post-treatment TTMV-HPV DNA metrics.

	**Recurrence**	**No recurrence**	**Predictive value**
Test positive	39	0	PPV=100% (91.0-100)
Test negative	7[A]	123	NPV=94.6% (90.7-98.5)
**Sensitivity and Specificity**	Sens.=84.8% (74.4-95.2)	Spec.=100% (97.0-100)	

[A] Only two of the seven patients with a discordant negative test had pretreatment TTMV-HPV DNA testing. Therefore, baseline detectability was not confirmed for five of the seven patients. These five patients had their HPV status confirmed by p16 IHC. The 95% confidence intervals (95% CIs) are shown in parentheses for respective values. 95% CIs calculated using the Wald method for metrics under 100% and the Wilson method for metrics equal to 100%.

Sens., Sensitivity; Spec. Specificity; NPV, Negative Predictive Value; PPV, Positive Predictive Value.
